# Assessment of inflammatory markers in late-onset schizophrenia

**DOI:** 10.1192/j.eurpsy.2025.2034

**Published:** 2025-08-26

**Authors:** V. Pochueva, I. Kolykhalov, L. Androsova

**Affiliations:** 1 FSBSI Mental Health Research Center, Moscow, Russian Federation

## Abstract

**Introduction:**

Previous studies have shown that neuroinflammation can play a significant role in the pathogenesis of schizophrenia. The search for inflammatory markers in late-onset schizophrenia are very important for diagnostics.

**Objectives:**

Determination of inflammatory markers in the peripheral blood plasma of patients with late-onset schizophrenia in relation to the clinical characteristics of patients.

**Methods:**

The study included 46 patients with schizophrenia aged 61 [56; 69] years (2 men and 44 women); the age of disease onset was 51 [45; 60] years. The severity of mental disorders was assessed using the PANSS scale, and cognitive impairment was assessed using the MMSE and MoCA scales. The control group consisted of 77 people comparable in age with the patients (p=0.16). A spectrophotometric method was used to determine inflammatory markers (enzymatic activity of leukocyte elastase (LE), functional activity of α1-proteinase inhibitor (α1-PI)). The protease inhibitory index (PII) was calculated - the ratio of LE and α1-PI activity, indicating the direction of the inflammatory process. Comparative data analysis was performed using the Statistica 10.

**Results:**

The 1^st^ cluster (22 patients (47.8%)) was characterized by a significantly increased α1-PI activity (p=0.000), decreased LE activity (p=0.000) compared to the control values, and, accordingly, a low PII value (p=0.000), which is an unfavorable prognostic factor for further development of the disease and response to therapy. A 28-day course of therapy with 1st and 2nd generation neuroleptics didn’t change the immunological parameters in patients of this cluster. In this cluster, a positive correlation was found between LE activity and scores on the MMSE (r=0.512, p<0.05) and MoCA (r=507, p<0.05) scales at the start of treatment, i.e. the lower score on the cognitive functioning scales and the more severe the disease correlate with lower the LE activity.

The 2^nd^ cluster (24 patients (52.2%)) was characterized by a significant increase in inflammatory markers, LE and α1-PI activity (p=0.000, p=0.000, respectively) in relation to the control parameters, while the PII value didn’t differ from the control. In this group, paranoid and schizotypal personality were significantly less common in the premorbid period (41.6% of cases), and formal thinking disorders were expressed to a lesser extent. No clinical and immunological relationships were revealed. A 28-day course of therapy influenced the change in immunological parameters towards their relative normalization.

**Conclusions:**

The obtained results confirm the involvement of the inflammatory link in the development of late-onset schizophrenia, as well as the heterogeneity of the patient group in terms of clinical and immunological parameters. Evaluation of the spectrum of inflammatory markers allows us to identify patients with an unfavorable course of the disease and resistance to therapy.

**Disclosure of Interest:**

None Declared

